# Erratum

**DOI:** 10.5935/abc.20190133

**Published:** 2019-07

**May issue of 2019, vol. 112 (5), pages 577-587**

In review article “Kidney Disease in Diabetes Mellitus: Cross-Linking between Hyperglycemia, Redox Imbalance and Inflammation”, consider Sandra Mary Lima Vasconcelos as the correct form for the name of the author Sandra Mary de Lima Vasconcelos.

**May issue of 2019, vol. 112 (6), pages 713-714**

In special article “Brazilian Society of Cardiology - The Women’s Letter”, consider Maria Christiane Valéria Braga Braile as the correct form for the name of the author Maria Cristiane Valeria Braga Braile.

